# Eosinophilic Ascites: A Rare Diagnosis With an Even Rarer Etiology

**DOI:** 10.7759/cureus.68511

**Published:** 2024-09-03

**Authors:** Devipriya Surapaneni, Bilal Azam, Sharath Chandra Dasi

**Affiliations:** 1 Internal Medicine, Saveetha Medical College and Hospital, Saveetha Institute of Medical and Technical Sciences, Chennai, IND; 2 Medical Gastroenterology, Saveetha Medical College and Hospital, Saveetha Institute of Medical and Technical Sciences, Chennai, IND

**Keywords:** corticosteroids, eosinophilic enteritis, peripheral eosinophilia, low saag ascites, eosinophilic gastrointestinal disorders

## Abstract

Eosinophilic ascites (EA) is a rare and often challenging clinical manifestation of eosinophilic gastroenteritis (EGE), a condition characterized by eosinophilic infiltration in various layers of the gastrointestinal tract. EA specifically involves the abnormal accumulation of eosinophils in the peritoneal cavity, which can lead to significant abdominal distension and discomfort. EGE is an inflammatory disorder that can affect the mucosal, muscular, or serosal layers of the gastrointestinal tract, primarily resulting from a combination of genetic predisposition, environmental triggers, and immune responses. This case report discusses a 39-year-old male who presented with persistent abdominal distension, significant weight loss, vomiting, and chronic diarrhea. Clinical evaluation revealed marked eosinophilia and EA, prompting a series of diagnostic tests to differentiate it from other potential causes such as parasitic infections and malignancies. Imaging studies indicated moderate ascites and intestinal wall thickening. The patient was diagnosed with eosinophilic enteritis, and treatment with corticosteroids led to substantial clinical improvement. This case highlights the diagnostic challenges and management strategies associated with both EA and EGE, emphasizing the importance of recognizing these rare manifestations of eosinophilic gastrointestinal disorders.

## Introduction

Eosinophilic enteritis (EE) is a rare gastrointestinal (GI) disorder characterized by diffuse or localized eosinophilic infiltration within the intestinal mucosa, leading to inflammation and associated symptoms, often associated with peripheral eosinophilia and diverse GI symptoms. EE was first described in medical literature in 1937 by Dr. Kaijser [1], a Swedish pathologist. He reported cases characterized by eosinophilic infiltration of the GI tract, with symptoms such as abdominal pain, diarrhea, and weight loss. This initial description laid the foundation for recognizing EE as a distinct clinical entity, and subsequent research has expanded the understanding of its pathophysiology, diagnosis, and treatment.

EE is divided into three primary subtypes according to the predominant GI tract layer affected by eosinophilic infiltration: mucosal, muscular, and serosal. This classification was initially delineated by Dr. Richard Klein and colleagues in their 1970 publication [2].

This condition is frequently linked with increased serum eosinophil levels and peripheral blood eosinophilia [3], and an increase in serum IgE levels, but this is not applicable to all cases. Eosinophilic gastrointestinal disorders (EGIDs) are thought to develop due to a mix of genetic and environmental factors. Around 10% of people with EGIDs have a notable family history of the condition. Additionally, strong evidence suggests an allergic basis for the disease, as roughly 75% of EGID patients have allergies [4].

Diagnosis can be challenging due to the nonspecific nature of symptoms and the need to exclude other conditions with similar presentations and involves several modalities, including a thorough clinical evaluation to assess symptoms, endoscopy for direct visualization of the GI tract, and biopsy to identify eosinophil infiltration in tissue samples. Imaging studies such as ultrasound or CT scans may be used to evaluate structural abnormalities, while food allergy testing and laboratory tests, including blood tests for eosinophilia, help identify potential triggers and confirm the diagnosis. Eosinophilic intestinal inflammation may also occur secondarily as a manifestation within the GI tract in association with conditions such as inflammatory bowel disease, autoimmune disorders, drug reactions, infections, and hypereosinophilia syndrome [5]. Therefore, it is essential to consider these differential diagnoses before establishing a diagnosis of EE.

The treatment of eosinophilic gastroenteritis (EGE) includes targeted dietary therapy, such as elimination diets to remove specific food antigens based on allergy testing, and the use of elemental diets to promote mucosal healing. Pharmacologic management typically involves corticosteroids [6] to reduce eosinophilic inflammation and immunosuppressive agents like azathioprine help manage chronic cases. Current literature supports the efficacy of dietary modifications and corticosteroid therapy in achieving symptom relief and histological remission, necessitating ongoing monitoring and adjustments in management to prevent the recurrence of symptoms.

## Case presentation

A 39-year-old male, farmer by occupation, presented to our outpatient department with a primary complaint of persistent abdominal distension for four months. The patient reported additional symptoms, including vomiting and pain in the abdomen over the last three months, with episodes occurring two to three times daily. The vomitus was non-bilious, non-projectile, and contained undigested food particles. The patient also experienced chronic diarrhea, characterized by semi-solid stools with increased frequency, devoid of hematochezia or melena. He noted significant unintentional weight loss, approximately 10 kg in the last two months, and a marked decrease in appetite. The patient's review of systems revealed no significant findings, specifically negative for fever, chills, night sweats, hemoptysis, dyspnea, chest pain, peripheral edema, or any previous history of GI bleeding. There was no record of recent medication intake, substance abuse, vaccinations, or travel history. He denied any known food or drug allergies.

On general examination, the patient exhibited pallor, and abdominal examination revealed significant distension consistent with massive ascites, accompanied by audible bowel sounds. There was no evidence of hepatosplenomegaly or palpable masses, and the abdomen was non-tender without signs of peritoneal irritation such as guarding or rigidity. The rest of the systemic examination showed no notable abnormalities.

Laboratory investigations showed normocytic anemia and marked eosinophilia. Peripheral blood smear analysis confirmed normocytic normochromic erythrocytes, mild anisopoikilocytosis, and an elevated eosinophil count with normal leukocyte and platelet morphology. Liver function tests were within normal limits. Abdominal ultrasonography revealed moderate ascites with no discernible organomegaly. Given the significant eosinophilia, absolute eosinophil count (AEC) and serum immunoglobulin E (IgE) levels were measured, showing elevated levels of 3470 IU/mL and 971 cells/µL, respectively (Table 1). The analysis of the ascitic fluid confirmed eosinophilic ascites and showed a low serum-ascites albumin gradient (SAAG) along with high protein levels (Table 2). Serial stool cultures, sensitivity tests, and routine stool examinations for ova and cysts were performed but returned negative results. In light of the patient’s occupation and clinical findings, including eosinophilic ascites, differential diagnoses included parasitic infections such as filariasis or strongyloidiasis. Serological testing for filariasis and a duodenal biopsy for strongyloidiasis were performed, both of which returned negative results. However, the patient was treated empirically with diethylcarbamazine and ivermectin for two weeks, targeting potential parasitic etiologies, without symptomatic improvement or resolution of ascites.

**Table 1:** Laboratory investigations on the day of admission and following two weeks of treatment with steroids. A significant improvement in eosinophilia is observed following initiation of treatment with steroids.

Labs	Day of admission	After 2 weeks of prednisolone treatment	Reference range	
Total leukocyte count (cells/cu.mm)	9010	5970	4000-10000	
Neutrophils (%)	45.7	76.8	40-80	
Lymphocytes (%)	13.2	19.9	20-40	
Monocytes (%)	4.1	2.0	2-10	
Eosinophils (%)	36.7	1.0	1-6	
Basophils (%)	0.3	0.3	<1-2	
Absolute eosinophil count (cells/cu.mm)	3470	180	20-500	
Serum IgE (IU/ML)	971.398	595.953		

**Table 2:** Ascitic fluid analysis. Ascitic fluid analysis showed a high serum-ascites albumin gradient (SAAG), low protein content, and a predominance of eosinophils. DC: differential count.

Labs	Value
Total count (cells/cu.mm)	1857
DC - Neutrophils (%)	4
DC - Lymphocytes (%)	6
DC - Eosinophils (%)	90%
Total protein	4.2
Albumin	1.8
Cytology	Category II - negative for malignancy
Culture sensitivity	No growth

**Figure 1:**
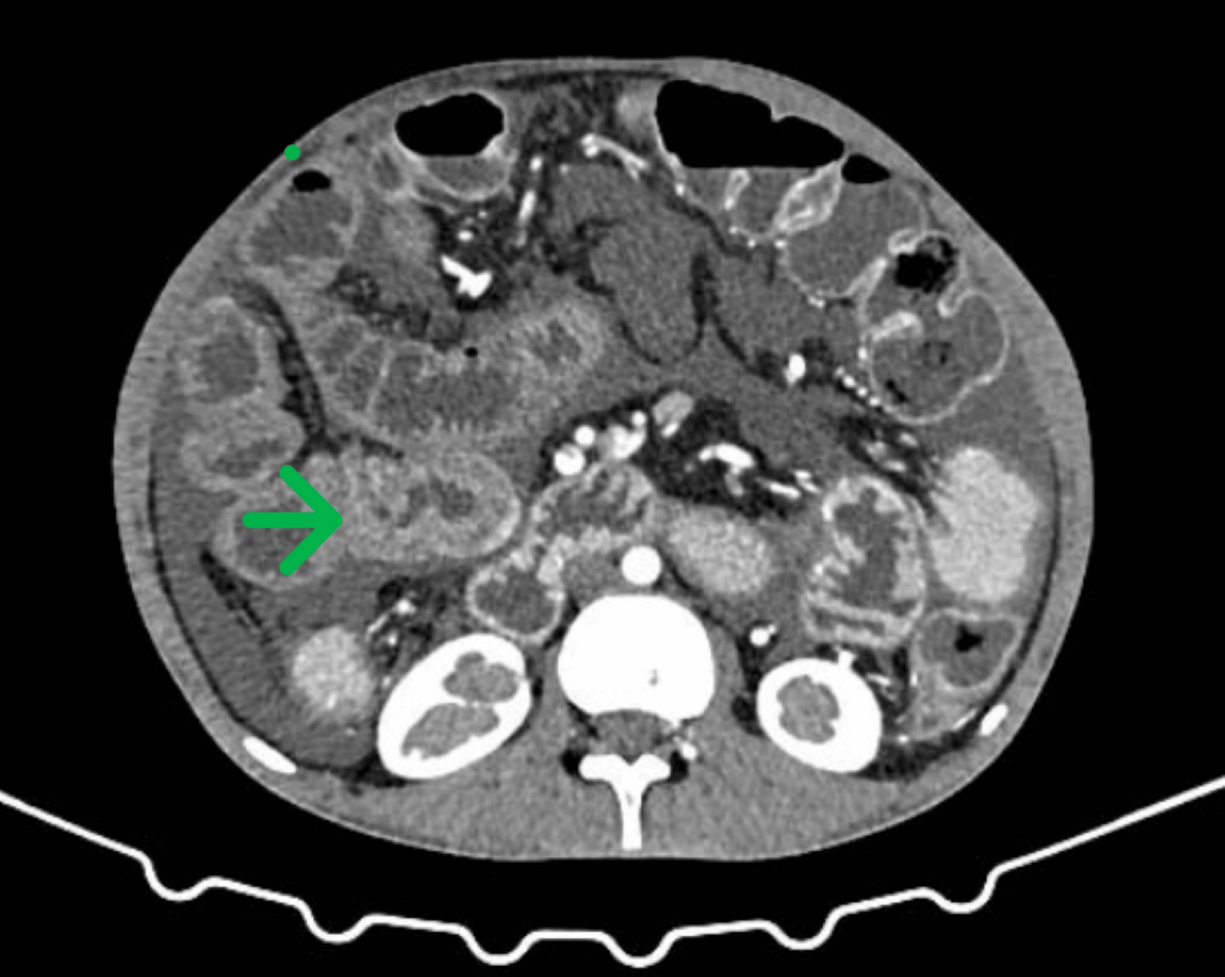
Triple-phase CT of the abdomen. Findings: Diffuse, circumferential, continuous, and long segmental, mural thickening was seen involving the ileal loops with a maximum thickness of 11 mm. Post-contrast imaging showed preserved mural stratification with mucosal and serosal enhancement and hypoenhancing submucosal edema was noted. No evidence of strictures/luminal narrowing was noted. No significant wall thickening was seen in large bowel loops. No obvious retroperitoneal lymphadenopathy. A severe amount of free fluid was seen involving perihepatic, hepatorenal, peri-splenic splenorenal, bilateral paracolic gutters, inter bowel spaces, pelvic spaces, and rectovesical pouch.

Upper GI endoscopy performed showed grade A distal esophagitis and diffuse mucosal erythema. Two biopsies from the duodenum and gastric mucosa were taken. Duodenal biopsy showed minimal lymphoplasmacytic infiltrate and no evidence of villous atrophy and intraepithelial lymphocytosis. While biopsy taken from gastric mucosa showed marked activity of *Helicobacter pylori* organisms, following confirmation of which empiric treatment for *H. pylori* was initiated. However, *H. pylori* gastritis was only an incidental finding and was not attributed to the clinical manifestations of the patient. An ileocolonoscopy was done and was normal. A CT enterography was done and the findings are presented in Figure 1.

**Figure 2:**
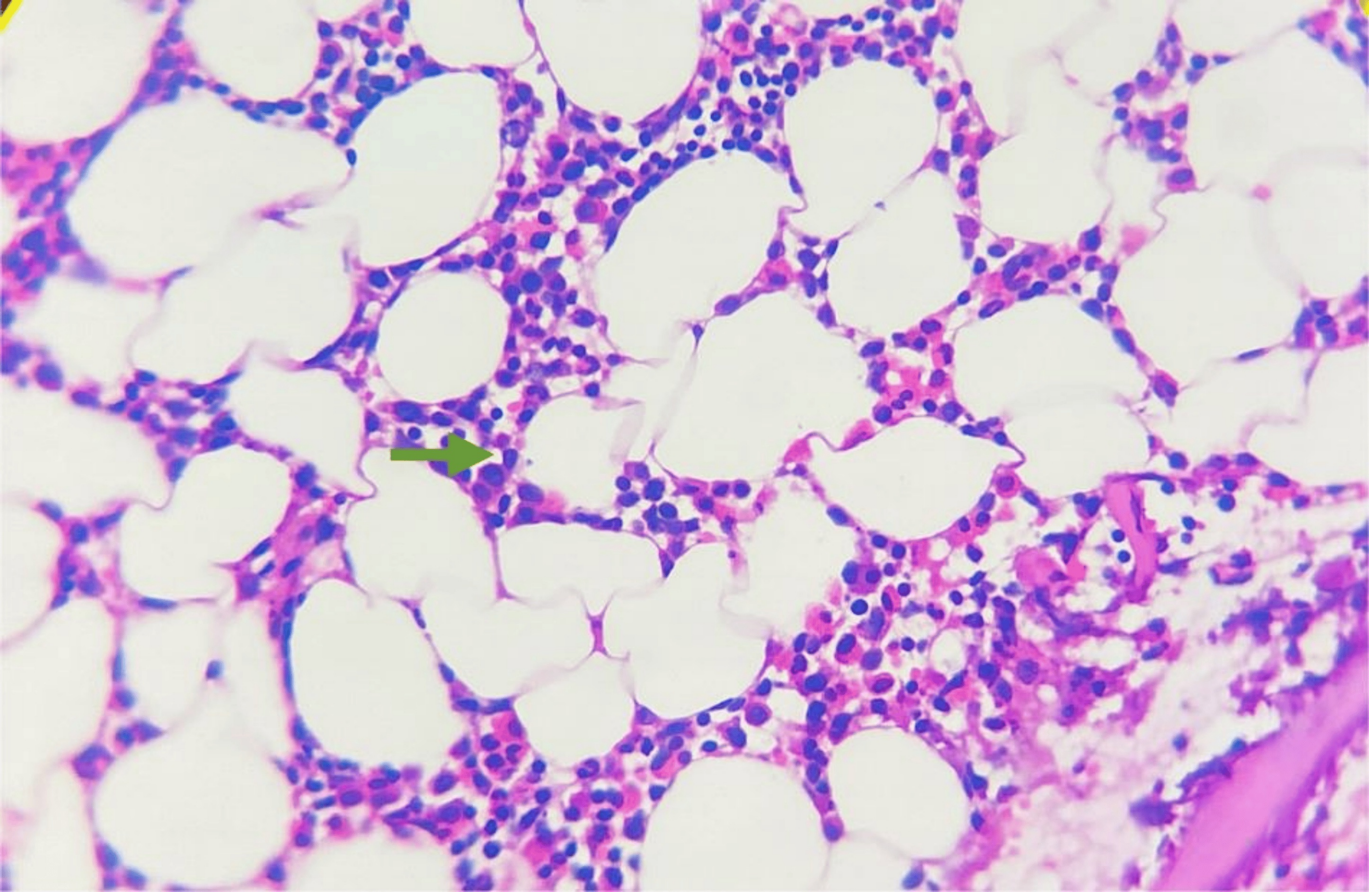
Bone marrow biopsy. Section showed a linear fragment of bony trabeculae enclosing mildly hypocellular marrow spaces. There was erythroid hyperplasia with normoblastic maturation. The myeloid series showed normal maturation with increased eosinophilic precursor. Megakaryocytes were adequate and normal in number. No granulomas/atypical cells were seen in the sections studied.

Given a high AEC and the presence of eosinophilic ascites, considering differential diagnoses such as inflammatory bowel disease, autoimmune vasculitis, hypereosinophilic syndrome (HES), and eosinophilic leukemia, further investigations were undertaken. To rule out celiac disease, tests for antibodies such as anti-tissue transglutaminase and anti-endomysial antibodies were conducted, both of which were negative. Both the antinuclear antibodies (ANA) and vasculitis profiles returned negative results, effectively ruling out any possible autoimmune etiology. A bone marrow aspiration and biopsy revealed erythroid hyperplasia with increased eosinophilic precursors and a mildly hypocellular marrow, characterized by normoblastic erythroid hyperplasia with eosinophilia (Figure 2). The findings from the bone marrow biopsy, along with the patient's clinical manifestations, did not align with the possible etiologies of HES or eosinophilic leukemia. Therefore, this necessitated further evaluation to explore other potential causes of eosinophilia.

**Figure 3:**
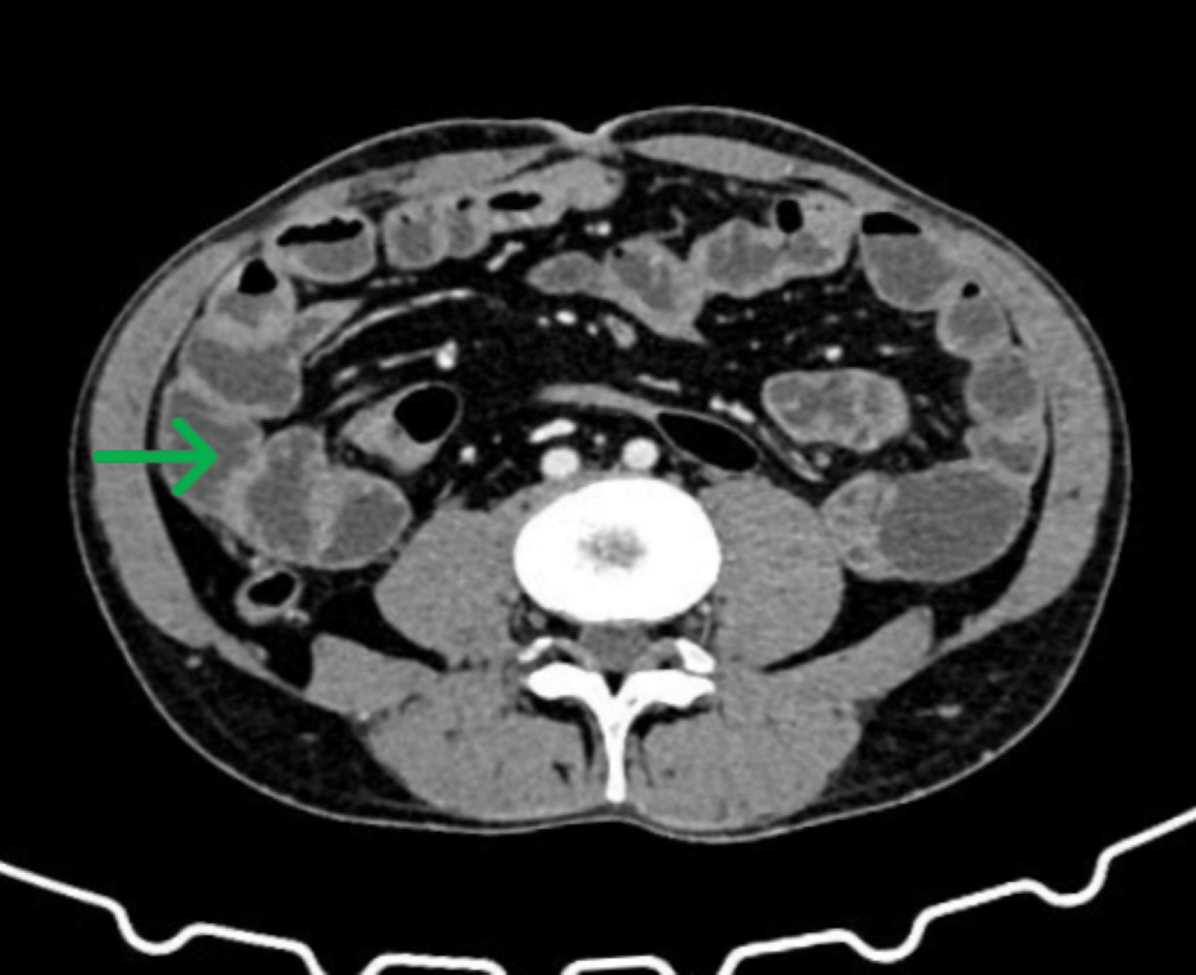
Triple-phase CT of the abdomen following one month of treatment with oral steroids. Findings compared to the previous study: No free fluid/collection was seen in the peritoneal cavity. The visualized bowel loops were not dilated.

Persisting symptoms and laboratory and radiological findings led to a diagnosis of eosinophilic gastroenteritis, characterized by elevated eosinophils and IgE levels unresponsive to anti-parasitic therapy, which is a diagnosis of exclusion. The patient was started on corticosteroid therapy, specifically prednisolone 40 mg daily. Follow-up at two weeks showed significant clinical improvement, with resolution of abdominal distension and normalization of eosinophil counts. Repeat measurements of AEC and serum IgE demonstrated a decreasing trend to 180 cells/µL and 595.95 IU/mL, respectively (Table 1). Following a month of corticosteroid therapy, the patient was transitioned to immunosuppressive therapy with azathioprine for long-term management. A month after the initiation of steroids, during follow-up, repeat CT enterography was done, which showed complete resolution of the bowel wall thickening (Figure 3). The patient was on regular follow-up and showed no recurrence of symptoms.

## Discussion

EGIDs are conditions marked by eosinophilic infiltration in the GI tract, including eosinophilic esophagitis (EoE) [7], EGE, and eosinophilic colitis. EGE was first documented in 1937 by Dr. Kaijser [1] as a benign condition of uncertain origin. It is characterized by recurring abdominal pain and diarrhea, with pathological features, including tissue edema, a dense eosinophilic infiltrate, and often, peripheral eosinophilia in most patients. As described by Klein et al., EE is classified into three subtypes based on the depth of eosinophilic infiltration within the GI tract: mucosal subtype, the most prevalent, involves eosinophilic infiltration of the mucosal layer, leading to abdominal pain and diarrhea. The muscular variant is defined by the presence of eosinophilic infiltration within the muscularis propria, resulting in bowel wall thickening, obstruction, etc. Lastly, the serosal subtype, the least common, affects the serosal layer and can cause eosinophilic ascites (EA) and abdominal distention [2].

Eosinophilic infiltration of the GI tract serves as a key histopathological feature of EGE. This infiltration is mediated by T-helper type 2 (Th2) cytokines, including IL-4, IL-5, and IL-13, which are essential in the disease's pathogenesis. Additionally, eotaxin and its receptor, CCR3 (cysteine-cysteine receptor 3), play a vital role in the migration of eosinophils to the GI tract [8]. It is a rare condition characterized by a variety of clinical manifestations that depend on the specific regions and layers of the GI tract affected.

The mucosal subtype of EE is the most prevalent and may manifest as abdominal pain, vomiting, diarrhea, and GI bleeding [9]. Features of malabsorption such as anemia and protein-losing enteropathy may be present. Those with muscular disease might present with bowel thickening and stenosis, and features of intestinal obstruction may be seen [10]. The serosal subtype, affecting approximately 10% of EGE cases, is identified by aseptic effusion with elevated eosinophils in ascitic fluid and typically presents with symptoms of ascites or peritonitis [11]. Our patient presented with abdominal distension, diarrhea, and vomiting as the primary clinical symptoms. The occurrence of ascites as the primary symptom of EE, especially in serosal type [2], is exceptionally uncommon, accounting for only 10-13% of cases. This was the chief complaint of our patient.

Diagnosing EA is challenging due to its broad differential diagnosis and nonspecific clinical presentation. Differential diagnoses for EA include parasitic infections such as *Strongyloides stercoralis* and *Toxocara canis*, spontaneous bacterial peritonitis (SBP), abdominal tuberculosis, disseminated hydatidosis, chronic pancreatitis, vasculitis (e.g., Churg-Strauss syndrome) [12], HES, various malignancies (e.g., ovarian cancer, Hodgkin's lymphoma, and peritoneal carcinomatosis), and inflammatory bowel diseases [13]. These conditions must be considered and ruled out to accurately diagnose EA. However, the incidence of EE as the cause of EA is quite significant. Given the patient’s occupation as a farmer and low socioeconomic status, parasitic infections were initially suspected; however, after a series of investigations, a diagnosis of EE was established.

A hallmark of EGE, peripheral eosinophilia [14], is present in a significant percentage of cases, often ranging from 20% to 80% of patients. Elevated IgE levels are also frequently seen [3], both of which were significant findings in our patient. Hypoalbuminemia and increased fecal fat excretion due to malabsorption are additional findings that are commonly present. In the diagnosis of EGE, both endoscopy and imaging studies play a crucial role. Endoscopic examination may reveal various findings such as erythematous, friable mucosa, ulcerations, pseudo polyps, and true polyps, although these are not definitive for EGE. Additionally, imaging studies such as ultrasound and CT scans complement the diagnostic process by identifying features like ascites, intestinal wall thickening, and mucosal fold thickening, which can guide biopsy efforts and detect complications such as obstruction [15].

Histopathology is key in diagnosing this condition with diagnostic thresholds ranging from over 20 to 30 eosinophils per high-power field (HPF) [16]. Laparoscopy remains the preferred method for obtaining a full-thickness specimen, offering the most accurate diagnosis, especially for the muscular and serosal types [17]. Based on the CT enterography showing ileal wall thickening, along with findings of EA, peripheral eosinophilia, and elevated IgE levels, we provisionally diagnosed the patient with EE and proceeded to treat him accordingly.

The treatment of EE is multifaceted and primarily depends on the severity of symptoms and the extent of disease involvement. Initial management often includes dietary modifications, such as elimination diets to identify and remove specific food allergens. Medications play a significant role, with corticosteroids (such as prednisone) [18] commonly used as the first-line treatment to reduce inflammation and eosinophilic infiltration. Topical corticosteroids like budesonide may also be employed with fewer systemic side effects. For patients who do not respond adequately to corticosteroids, immunosuppressive agents, such as azathioprine [19] or methotrexate, may be considered. Emerging treatments with biologics targeting interleukin-5 (IL-5), such as mepolizumab [20] and reslizumab, have shown promise in reducing eosinophil levels. Additionally, management of any complications, such as bowel obstruction, may necessitate surgical intervention. In our case, an excellent response to steroids and azathioprine was seen.

In reviewing the existing literature on EE and EA, several notable case reports provide valuable insights into this rare condition (Table 3).

**Table 3:** Summary of clinical case reports on eosinophilic enteritis and ascites, highlighting findings and treatments. AEC: absolute eosinophil count; SAAG: serum-ascites albumin gradient; IgE: immunoglobulin E.

No.	Author	Clinical findings	Imaging	Laboratory findings	Treatment
1	Tahir et al. (2024) [21]	Abdominal pain, vomiting, loose stools, dysphagia (solids and liquids)	Abdominal CT: moderate ascites along with notable diffuse thickening of the jejunal mucosa. The right upper quadrant ultrasound indicated the presence of ascitic fluid surrounding the liver	AEC = 7.98 × 109/L. High SAAG low protein ascitic fluid with 92% eosinophils. Laparoscopic full-thickness jejunal biopsy demonstrated a significant increase in eosinophils within the lamina propria	Intravenous followed by oral methylprednisolone. Lactose and gluten-free diet changes
2	Galere et al. (2022) [22]	Worsening abdominal pain, nausea, vomiting, tender abdomen with bulging flanks	CT scan: absence of visceral lesions	AEC = 8·94 × 109/L, raised IgE concentration (208 IU/mL), elevated CA125 concentration (83 units/mL)	Oral methylprednisolone
3	López-Sáez et al. (2021) [23]	Recurrent seasonal ascites, abdominal distension, diarrhea, nasal conjunctivitis, and bronchial symptoms coinciding with spring months	Ultrasound and computed axial tomography revealed free abdominal fluid	Peripheral blood eosinophilia (2200/μL), 95% polymorphonuclear eosinophils in ascitic fluid, elevated total serum IgE (>390 kU/L), eosinophilic infiltration in the lamina propria of the duodenum (40 eosinophils/hpf).	Oral corticosteroids for severe episodes, bland diet for milder episodes, dietary exclusion of trigger foods
4	Kim et al. (2019) [24]	Diarrhea, abdominal pain, recent influenza A virus infection	Abdominal CT: massive ascites, bowel wall thickening in the ileum & ascending colon wall	Eosinophil count = 6,351 cells/mm³. Serum IgE = 375.7 IU/mL. Eosinophilic infiltration in the ileum	Oral prednisolone, dietary exclusion (trigger foods)
5	Letrán et al. (2018)[25]	Epigastric pain, abdominal distention, nausea, vomiting	Abdominal CT: moderate ascites. Upper GI endoscopy revealed no macroscopic abnormalities	Eosinophil count = 8.94 × 10⁹/L. Serum IgE = 208 IU/mL. Eosinophilic infiltration in the lamina propria of the duodenum (35 eosinophils/hpf)	Oral prednisone, wheat-free diet

## Conclusions

In conclusion, this case report highlights the challenges of diagnosing eosinophilic enteritis particularly when it presents with eosinophilic ascites. Despite the initial consideration of other causes due to the patient's background and presenting symptoms, a thorough diagnostic workup ultimately led to the identification of EE. The patient's response to steroid therapy and azathioprine underscores the effectiveness of these treatments in managing the condition. EGE is a complex disorder that requires careful consideration of dietary triggers, clinical symptoms, and histopathological findings for accurate diagnosis and successful management. This case highlights the need for comprehensive assessment, including both clinical evaluations and appropriate imaging studies, to achieve a successful outcome in patients with eosinophilic gastrointestinal disorders. Regular follow-up is important to monitor the patient's progress and adjust treatment as necessary.
